# Understanding the Differential Impact of Vegetation Measures on Modeling the Association between Vegetation and Psychotic and Non-Psychotic Disorders in Toronto, Canada

**DOI:** 10.3390/ijerph18094713

**Published:** 2021-04-28

**Authors:** Abu Yousuf Md Abdullah, Jane Law, Zahid A. Butt, Christopher M. Perlman

**Affiliations:** 1 School of Public Health and Health Systems, University of Waterloo, Waterloo, ON N2L 3G1, Canada; jane.law@uwaterloo.ca (J.L.); zahid.butt@uwaterloo.ca (Z.A.B.); chris.perlman@uwaterloo.ca (C.M.P.); 2 School of Planning, University of Waterloo, Waterloo, ON N2L 3G1, Canada

**Keywords:** vegetation, mental health, spatial modeling, psychotic, non-psychotic, enhanced vegetation index, normalized difference vegetation index, soil-adjusted vegetation index, Bayesian, random forest

## Abstract

Considerable debate exists on whether exposure to vegetation cover is associated with better mental health outcomes. Past studies could not accurately capture people’s exposure to surrounding vegetation and heavily relied on non-spatial models, where the spatial autocorrelation and latent covariates could not be adjusted. Therefore, a suite of five different vegetation measures was used to separately analyze the association between vegetation cover and the number of psychotic and non-psychotic disorder cases in the neighborhoods of Toronto, Canada. Three satellite-based and two area-based vegetation measures were used to analyze these associations using Poisson lognormal models under a Bayesian framework. Healthy vegetation cover was found to be negatively associated with both psychotic and non-psychotic disorders. Results suggest that the satellite-based indices, which can measure both the density and health of vegetation cover and are also adjusted for urban and environmental perturbations, could be better alternatives to simple ratio- and area-based measures for understanding the effect of vegetation on mental health. A strong dominance of spatially structured latent covariates was found in the models, highlighting the importance of adopting a spatial approach. This study can provide critical guidelines for selecting appropriate vegetation measures and developing spatial models for future population-based epidemiological research.

## 1. Introduction

The effect of vegetation on mental health is a topic of considerable debate in recent years. Several carefully designed studies have obtained contradictory results while assessing the role of vegetation in improving mental health conditions. Although evidence suggests that vegetation-covered areas can have an ameliorating effect on people’s psychological well-being [1], other studies have reported that vegetation is either weakly associated with mental health or does not have any consistent and significant effect [2,3]. 

Contrary to traditional beliefs, good mental health now extends beyond the state of absence of mental disorders. According to the World Health Organization [4], “mental health is a state of well-being in which the individual realises his or her own abilities, can cope with the normal stresses of life, can work productively and fruitfully, and is able to make a contribution to his or her community”. Common mental health disorders, which may induce poor mental health conditions in the urban population, can be broadly categorized into psychotic and non-psychotic disorders [5,6,7]. Psychotic disorders are characterized by the loss of touch with reality, leading to delusions, hallucinations, and disorganized thinking and behavior [6,8]. In contrast, non-psychotic disorders affect an individual’s state of mind, behavior, and ability to think and feel, which may manifest in the form of depressive, anxiety, dissociative, and personality disorders [9,10]. Several studies have reported an increased prevalence of psychotic disorders in urban areas and found that urban stressors elevate the risk of developing psychotic disorders [11,12]. Similarly, non-psychotic disorders were found to be the most prevalent among all the types of mental health disorders in Ontario, Canada [9], a province that contains five of the fastest growing urban areas in the country [13]. 

Therefore, given the global increase in urbanization and the subsequent loss in vegetation-covered areas [14,15], understanding whether reduced vegetation cover can act as a putative risk factor for mental health disorders, such as psychotic and non-psychotic disorders, has become an issue of critical importance [1]. Past studies indicate that urbanization is predominantly followed by the loss of surrounding vegetation [14,15], which may be a potential threat to urban residents’ mental health and well-being [16]. For example, White et al. (2013) studied the relationship between urban green space and mental distress and found that individuals living in areas with more urban green space reported having lower mental distress [16]. Additionally, past studies reported that even simple walks within vegetation-rich areas such as parks or streets could help mitigate cognitive fatigue and depression in adults [17,18]. Similar benefits of the vegetation and natural environment were found for children with attention deficit hyperactivity disorder (ADHD) in urban areas. A twenty-minute walk in an urban park (having a prominent presence of vegetation) yielded elevated attention performance for these children compared to walks in downtown and residential areas [19]. 

Unfortunately, some methodological constraints in studying vegetation and mental health are rarely addressed in epidemiological studies. The first challenge stems from the fact that characterizing or defining “vegetation” in urban areas can be extremely difficult, since vegetation comes in different forms. Ideally, vegetation can be defined as a collection of plants that includes, but is not limited to, tall trees in protected areas, shrubs and bushes in parks, and ornamental plants in gardens and on rooftops [20]. Therefore, the association between vegetation and mental health could show differential sensitivity depending on the type of vegetation measure [1]. The type of vegetation measure determines whether all vegetation forms in an urban area are considered during the study. Ideally, the best vegetation measure will be the one that can effectively capture people’s perception of and interactions with surrounding greenness in the study area [1,21,22]. 

In this regard, remote sensing- or satellite-based indices could be used to measure vegetation cover in urban areas. Several prominent indices are available, which include the normalized difference vegetation index (NDVI) [23], enhanced vegetation index (EVI) [24], and the soil-adjusted vegetation index (SAVI) [25]. All these indices utilize the type and intensity of electromagnetic waves reflected from vegetation cover to detect vegetation in an area [26]. Rugel et al. (2017) discussed that NDVI could be used to effectively characterize vegetation in population-level mental health research [27]. However, Markevych et al. (2017) noted that highly sensitive vegetation indices such as EVI and SAVI could be more useful compared to the conventionally used NDVI and other polygon-based measures of vegetation [1]. Unlike NDVI, the computation process of these indices adjusts for atmospheric disturbances, background canopy cover, and spurious soil brightness [23,24,26] and thus can better capture the vegetation signals registered in satellite images. However, despite their computational differences, all three indices are able to measure both the areal extent and quality or the biomass vigor of vegetation cover [1,25,28]. In contrast, the commonly used polygon- and area-based vegetation measures [1,29,30] can only provide information on the areal extent and cannot measure the quality of the vegetation cover.

The second major methodological challenge stems from the complexities in selecting an appropriate statistical technique to study the association. The distributions of mental health disorder cases and vegetation cover are both spatially structured due to their varying levels of distribution in space [31,32]. Consequently, the use of non-spatial models such as multiple linear or logistic regression models, which assume structural stationarity of the dependent and independent variables over space, can be a gross oversimplification of the real-life scenario [33], especially when mental health disorder cases or vegetation cover are spatially autocorrelated. Spatial autocorrelation refers to a systematic spatial variation and originates when the observations (for example, the mental health disorder cases) in an area are affected by the observations of the neighboring areas [34]. Spatial autocorrelation may violate the core model assumptions of commonly used statistical models, which treat observations and residual errors as independent [35,36]. Hence, spatial autocorrelation or spatial dependence may impair the estimation of beta (β) coefficients and the accuracy of a significance test.

Furthermore, not all spatial techniques can measure the effects of latent covariates in the regression models [37]. These latent covariates are unmeasured socioeconomic and cultural factors that affect the distribution of mental health disorder cases in the study area [38]. If unadjusted, these covariates can obscure vegetation’s actual effect on mental health and can confound the results. Additionally, some frequentist spatial models, such as the spatial error and spatial lag models, consider spatial effects as a nuisance and adjust them accordingly during the estimation of β coefficients [36,37]. As a result, these models cannot separately measure the relative contributions of spatial and non-spatial latent covariates in the data generating process [38,39,40], which are essential for understanding the nature of risk factors and devising targeted interventions.

Contrary to these frequentist approaches, Bayesian spatial modeling (BSM) can be applied to adjust for the spatial autocorrelation and capture the spatial structure of the covariates through the integration of a spatial random effect term ($s_{i})$ in the models. Additionally, any overdispersion in the count data of mental health disorders could be adjusted using a non-spatial or spatially unstructured random effect term ($u_{i}$) [39,40]. Furthermore, epidemiological studies are often interested in the area-wise relative risk of mental health disorders, which cannot be estimated precisely when the population size is too small or large. For example, extreme relative risk values are commonly associated with areas having small populations, while statistically significant relative risk values are associated with areas with large populations [40]. These artifacts owing to variations in the population size can also be adjusted in Bayesian models through the process of “borrowing” information from adjacent areas. Under this process, the models carry out statistical smoothing and incorporate the prior information (evidence from the data of surrounding areas) and the observed data of the area for which the risk will be estimated. Therefore, any statistical artifacts such as small data counts and large variations in sample size or study populations are inherently adjusted within the Bayesian models [39,40,41]. Consequently, through the application of BSM, it is possible to accurately identify areas with high risk of mental health disorders due to the influence of putative risk factors like low vegetation content.

Although some studies have attempted to understand the association between vegetation and mental health [27,42], there is a paucity of studies that have incorporated spatial statistics to understand how the selection of various vegetation measures could influence the association between vegetation and mental health. Hence, to address the research gap, we developed a spatial study based on the hypothesis that the type of vegetation measure could affect the significance of the association between vegetation and psychotic and non-psychotic disorder cases. In order to test this hypothesis, we used remote sensing and machine learning techniques to develop three vegetation indices (EVI, SAVI, and NDVI) and an area-based vegetation measure from satellite images. We also retrieved the conventionally used tree cover dataset from the Toronto Open Data Portal to compare with the satellite-based measures. Finally, we modeled the relationship between the two prevalent mental health disorders in urban areas, psychotic and non-psychotic disorders, and each of the vegetation measures using the Bayesian spatial modeling approach. The results were then compared in terms of the significance of association, relative risk values and the influence of spatial and non-spatial latent covariates in the Bayesian models. Thus, the study aimed to answer two specific research questions:

1. Can the type of vegetation measure affect the significance of the association between vegetation and psychotic and non-psychotic disorders?
2. What type of vegetation measures are associated with these two types of mental health disorders?

## 2. Materials and Methods

### 2.1. Study Area

This study focused on the city of Toronto, with a population of 2,731,571 in 2016 (Canadian census, 2016). The study was conducted at the neighborhood level, and all 140 neighborhoods were considered in the analysis. The neighborhoods were defined by the Social Policy Analysis and Research Unit in the Social Development and Administration Division of the City of Toronto [38]. These neighborhoods are geographic units created for planning and service delivery purposes by aggregating the Statistics Canada Census Tracts into meaningful spatial units [43]. The city of Toronto is one of the most urbanized and populous cities in the province of Ontario, Canada, and due to the high urbanization rate, the built environment is becoming the dominant land cover type in the area. The proliferation of the built environment is believed to have drastically reduced the soil volume available for the growth of small vegetation and has also decreased the aerial space for the growth and expansion of large trees. The increase of built surfaces has led to a rise in non-permeable surfaces and an increase in the ground salinity level due to the use of de-icing salt on the roads during the winter season. The environmental impacts of these changes are the dehydration and death of natural flora [44]. 

### 2.2. Data Preparation

#### 2.2.1. Mental Health Disorders

The mental health disorder data, covering the period from 1 April 2015 to 31 March 2016 (fiscal year 2015), were retrieved from the Ontario Community Health Profiles Partnership database [45]. The dataset was a part of the study “Enrollment, Access, Continuity and Mental Health Gaps in Care (ICES Project No. 2018 0900 992 000)”, which was supported by the Institute for Clinical Evaluative Sciences (ICES), funded by the Ontario Ministry of Health and Long-Term Care (MOHLTC). For this study, we extracted only the count data for psychotic and non-psychotic disorder cases, which are the two prevalent mental health disorders in the study area. Further details on the data can be found in the Supplementary Materials. 

The observed counts on psychotic and non-psychotic disorder cases for both sexes (males and females) and ages 0+ years were used as outcome variables to analyze how indices- and area-based measures of vegetation can impact the association between vegetation cover and psychotic and non-psychotic disorders among gender and age groups. The population at risk was also accounted for in the BSM models by estimating the expected counts of psychotic and non-psychotic disorders. The original data were divided into “enrolled” and “non-enrolled” categories. The term “enrolled” relates to the primary care enrollment models found in the Client Agency Provider Enrollment (CAPE) tables. The CAPE tables are used to identify patients enrolled in different primary care models over time [9]. As the target was to model the distribution of the mental health disorder cases regardless of the patient’s enrollment statuses in the primary care models, enrolled and non-enrolled cases were combined for the analyses. The final dataset contained counts of all the Ontario permanent residents with an Ontario Health Insurance Plan (OHIP) and having OHIP claims for the mental health conditions listed in Table 1 [9,45].

The expected counts of the mental health disorders were derived separately for the psychotic and non-psychotic cases using an indirect (internal) standardization method. The expected counts of psychotic or non-psychotic disorders correspond to the overall rate of psychotic or non-psychotic disorder cases multiplied with the residential population of each of the neighborhoods. This process involved applying the sex-specific rates to the population structure of each neighborhood and calculating the expected number of cases. As the data provider did not use the age of the individuals to group the data for psychotic and non-psychotic disorder cases, only the sex-specific rates could be used to estimate the expected counts of psychotic and non-psychotic disorder cases. 

**Figure 1:**
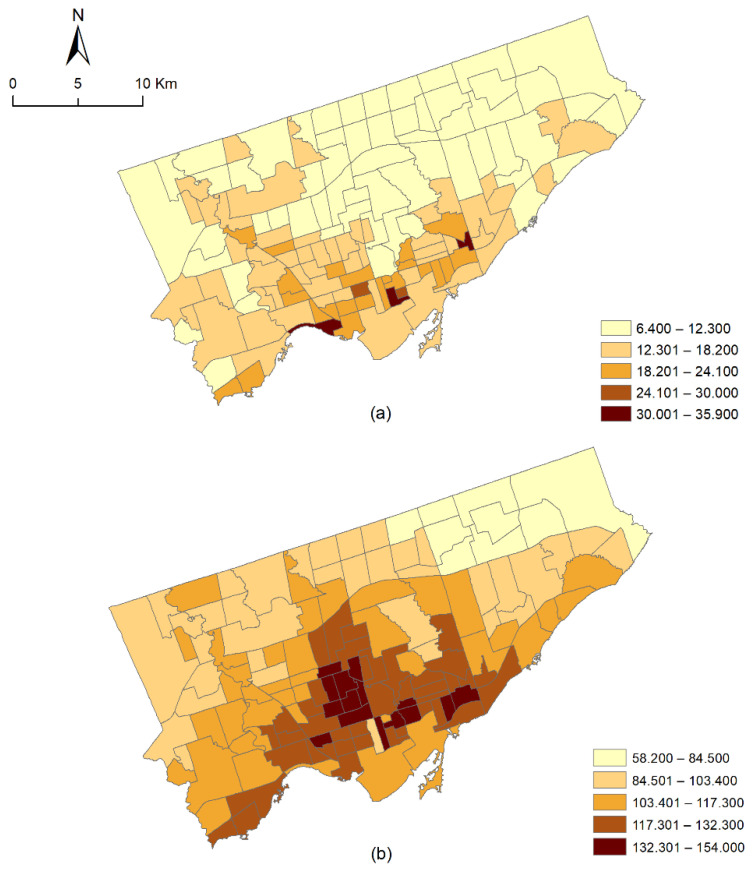
The age and sex standardized rates of (**a**) psychotic and (**b**) non-psychotic disorders.

The quantile maps in Figure 1 show that both the high and low rates of mental health disorders are concentrated in particular parts of the Toronto area, suggesting that the mental health disorder cases could be spatially autocorrelated. Since it is necessary to confirm the presence or absence of spatial autocorrelation in the data prior to selecting an appropriate modeling technique, the global Moran’s I test was carried out using the GeoDa software (https://geodacenter.github.io/, accessed on 25 September 2019) to test for spatial autocorrelation. The results suggested the presence of significant spatial autocorrelation in the data, which indicated that a spatial modeling technique would be essential to study the association between vegetation and the different types of mental health disorder cases. The details of the spatial autocorrelation test can be found in the Supplementary Materials. 

#### 2.2.2. Landsat 8 Satellite Images

Three satellite images of the Landsat Operational Land Imager and Thermal Infrared Sensor (OLI-TIRS) or Landsat 8 were retrieved from the United States Geological Survey’s (USGS) EarthExplorer data repository [46]. A search criterion of less than 10% cloud cover was used to exclude the images with a considerable presence of clouds, since cloud cover in satellite images can considerably influence the calculation of the vegetation indices [26]. Three images, having an average cloud cover of 2.67% and a spatial resolution of 30 m, were required to cover the study area. Two of these images were acquired on 20 May 2016, and the third image was captured on 14 June 2016. The year 2016 was selected to be consistent with the data period of the mental health disorder dataset. Similarly, the May and June months were chosen to estimate the vegetation content of the spring–summer seasons, because the vegetation densities during these months become stable after a cold, snowy winter and before a chilly fall season. 

Radiometric corrections were conducted by converting the raw digital numbers to the top-of-atmosphere (TOA) reflectance [47]. Furthermore, atmospheric corrections were applied to remove any haze from the images through the identification of the darkest pixel value in each band and subtracting this value from every pixel in the satellite image [48]. Finally, the radiometrically and atmospherically corrected images were mosaiced and cropped using the boundary of the city of Toronto to produce a single image for the analysis. 

#### 2.2.3. Construction of the Vegetation Indices

The three vegetation indices, EVI, NDVI, and SAVI, were generated using the processed Landsat 8 image for Toronto. The details of the vegetation indices and computational formulas used in this study can be found in the Supplementary Materials. The *Raster Calculator* in ArcMap 10.7 software (https://desktop.arcgis.com/en/arcmap/, accessed on 25 November 2019) from ESRI (Redlands, CA, USA) was used to perform the band operations to produce the rasters of vegetation indices. The formulas for the computation of these vegetation indices were obtained from the USGS websites for each of the indices [23,24,49]. These three indices are computed from different ranges of wavelengths in the electromagnetic spectrum (referred to as bands), reflected from the vegetation surface and received by the satellite. The reflectance of these bands, in turn, is governed by factors such as the type of plant, water retention capacities of the tissues, chemical and morphological characteristics of the leaves, and the level of photosynthetic activities in the plant [50,51,52]. 

The NDVI raster was used to extract the vegetation-covered areas and to mask out the non-vegetation features like water bodies, bare soil, and built-up surfaces in all three vegetation rasters. This process was necessary to remove the negative values representing the non-vegetation features in the vegetation indices [1]. Finally, the mean values of the indices for each neighborhood were extracted using the *Zonal Statistics* tool in ArcMap 10.7. 

#### 2.2.4. Developing the Land Use/Land Cover Model Using the Random Forest Ensemble

A land use/land cover (LULC) model was developed to estimate the percentage of vegetation cover in the Toronto area. This LULC model was developed to generate an area-based measure of vegetation through the application of an advanced machine learning ensemble, which could be compared with the area-based measure of vegetation derived using the automated extraction of features and custom-made procedures (such as the tree cover dataset from the Toronto Open Data Portal). Furthermore, this LULC model also allows a comparison between the vegetation indices that are able to estimate the plant biomass vigor and the area-based measures that are simply able to measure the areal extent of vegetation cover. 

The Random Forest (RF) classifier was chosen to develop the LULC model. This is because RF is one of the most powerful machine learning classifiers to date and can handle the classification of both multispectral and hyperspectral satellite images in noisy, unbalanced and non-linear data settings [53,54,55]. The use of the RF ensemble ensures significantly better classification accuracies, especially when the classification for areas such as Toronto could be heavily complicated due to the mixture of built environment and natural features [56,57]. For example, it would be challenging for an algorithm to distinguish between a green-colored building and a tree with a large green canopy. 

This study employed the “randomForest” package in R for the classification process [58]. Google Earth aerial imagery for the Toronto area in 2016 was used to generate the training data to be later used in the RF algorithm. On-screen visual interpretation and the NDVI image were used to assist the generation of the training dataset for the vegetation class. A total of 400 training data points were generated to develop the LULC model. A uniform number of training data (100 per class) was maintained for all the four land cover classes listed in Table 2. Furthermore, to ensure a homogenous distribution of the training dataset over the study area, all the parts (north, south, east, and west) of the study area were equally considered for generating the training data. Finally, to assess the accuracy of the developed model, 25% of the training data was retained for accuracy assessments, which means 75% of the data was used for training the RF model.

A different number of input features (*mtry*) and the number of decision trees (*ntree*) parameterization were performed to inspect the out-of-bag (OOB) error rates. Finally, an RF model was trained with a *ntree* and *mtry* setting that contributes to the lowest OOB error rate. Consequently, the final RF model on the training data was trained using the number of decision trees (*ntree*) as 500 and the number of input features (*mtry*) as 3. This trained model was then used for predicting LULC classes in the satellite image. Lastly, the vegetation-covered areas were extracted from the LULC raster and the “*Tabulate Intersection*” tool in the ArcMap software was used to estimate the percentage of area covered by vegetation (hereinafter referred to as Veg_RF) in each neighborhood.

#### 2.2.5. Processing the Tree Cover Dataset from the Open Data Portal

The tree cover dataset was retrieved from the “Treed area” data in the Toronto Open Data Portal. The City of Toronto’s Open Data Portal is a public data repository that allows developers, students, and researchers to easily avail spatial and non-spatial datasets related to the functioning of the city. The tree cover dataset was developed via automated extraction from aerial Light Detection and Ranging (LiDAR) using custom-developed procedures and open-source tools [59] and was a representation of the physical features (trees) that were visually identifiable in an aerial photograph. The data was downloaded in a shapefile (.shp) format and was converted to the Universal Transverse Mercator (UTM) projection for further use. The percentage of area covered by trees in each neighborhood (hereinafter referred to as Tree_OD) was estimated using the “*Tabulate Intersection*” tool in ArcMap.

#### 2.2.6. Adjusting for Potential Confounders

Socioeconomic factors can profoundly impact the mental well-being of people of all ages and sexes [60,61,62]. For example, psychotic disorders like schizophrenia were found to be more prevalent in lower than in higher socioeconomic groups [61]. Similarly, past research found association between low socioeconomic status and psychotic disorders, which may even lead to mood disorders and self-harm in adults [63]. Factors such as material deprivation, residential instability, dependency, and ethnicity have well-documented influences on mental health conditions [64,65,66,67]; therefore, these socioeconomic covariates were retrieved from the Ontario Marginalization Index (OMI) [68] and adjusted as potential confounders in the models. The OMI is an area-based index that highlights the differences in marginalization between geographic areas in Ontario and is comprised of four dimensions: material deprivation, residential instability, dependence, and ethnic concentration. The weighted average scores for these four variables were used, with a high score representing high material deprivation, residential instability, dependence, or ethnic concentration. In this regard, high ethnic concentration implies a high concentration of recent immigrants and visible minorities [68]. 

In order to avoid multicollinearity due to the addition of the four OMI variables, Pearson correlation coefficient [69] and multicollinearity [70] tests were conducted to check for significant correlations and whether these dimensions could be linearly predicted from one another. The results of the tests indicate that the OMI variables do not demonstrate sufficient inter-correlations and multicollinearity. Therefore, all four of the variables could be included in a regression model. The details of the socioeconomic covariates and the correlation and multicollinearity tests can be found in the Supplementary Materials.

In addition to the socioeconomic factors, substance use disorder may have a marked effect on the mental well-being of people [71,72]. To adjust for the effect of substance use, the age and sex standardized rate of substance use disorders (both sexes, 0+ age, and per 1000 population) was retrieved from the Ontario Community Health Profiles Partnership database [9,45] and added as a potential confounder in the Bayesian models. The summary statistics of the variables used in this study are given in Table 3.

#### 2.2.7. Bayesian Spatial Modeling

The association between vegetation and mental health was analyzed using the Bayesian spatial modeling (BSM) technique. For this process, the observed counts, $O_{ik},$ of the mental health disorder *k* in neighborhood *i* was assumed to follow a Poisson distribution. In this study, *k* = 1 or 2, representing psychotic and non-psychotic disorders, respectively. Similarly, *i* = 1, 2,…n, where *n* is the total number of neighborhoods in the city of Toronto (*n* = 140). Hence, Equation (1) can be used to define the distribution of the observations.

(1) $$O_{ik}\sim \;Poisson\left(\lambda_{ik}\right)$$

where $\lambda_{ik}$ represents the expected value of the mental health disorder *k* in the neighborhood *i*.

Equation (1) could be further modified to Equations (2) and (3). Equation (2) shows that the observed count of a particular mental health disorder in a neighborhood is a product of the unknown area-specific relative risk of the disorder,$\;r_{ik},$ and the expected count, $E_{ik}$. The $E_{ik}$ for each neighborhood was calculated earlier using the overall rate of the disorder, *k*, multiplied with the residential population of each of the neighborhoods. In contrast, the $r_{ik}$ was estimated using the Bayesian models. 

Therefore,

(2) $$\lambda_{ik}=E_{ik}\times r_{ik}=E_{ik}r_{ik}$$

Applying logarithm to both sides of Equation (2),

(3) $$\log\;[\lambda_{ik}]=\log\;[E_{ik}]+\log\;[r_{ik}]$$

The unknown area-specific relative risk can be assumed to be associated with the attributes of the population (socioeconomic) and environmental characteristics, or both [40]. As a result, for this study, the $r_{ik}$ can be substituted by the risk owing to the area-specific variations in the vegetation cover.

(4) $$\log\;[\lambda_{ik}]=\log\;[E_{ik}]+\beta_{0}+\beta_{1}X_{1i}$$

where $X_{1i}$ is the variable for vegetation measure (EVI, NDVI, SAVI, Veg_RF, or Tree_OD).

Additionally, as noted earlier, the socioeconomic conditions (represented by the four OMI variables) and the rate of substance use can influence the observed counts of psychotic and non-psychotic disorders in an area. Consequently, the material deprivation $\left(X_{2i}\right)$, ethnic concentration $\left(X_{3i}\right)$, residential instability $\left(X_{4i}\right)$, dependency $\left(X_{5i}\right)$, and the age and sex standardized rate of substance use disorders $\left(X_{6i}\right)$ were added into the model as potential confounders. Hence, Equation (4) gives

(5) $$\log\;[\lambda_{ik}]=\log\;[E_{ik}]+\beta_{0}+\beta_{1}X_{1i}+\beta_{2}X_{2i}+\beta_{3}X_{3i}+\beta_{4}X_{4i}+\beta_{5}X_{5i}+\beta_{6}X_{6i}$$

Although Equation (5) gives the desired model, several problems need to be considered before finalizing the model equation. First, the overdispersion in count data of the observed cases of mental health disorders is adjusted. One of the core assumptions of the Poisson model is that $Var\;\left[O_{ik}\right]=\lambda_{ik}$, where Var [] represents the variance. This implies that for a proper Poisson model, the mean of the observations needs to be equal to the variance of the observations. However, during overdispersion, $Var\;\left[O_{ik}\right]>\lambda_{ik}$, which means the variance in the count data is higher than expected by the modeled Poisson distribution. This overdispersion stems mainly from the heterogeneity in the individual-level risk of contracting the different types of mental health disorders, which translates to the heterogeneity observed in the count data of the psychotic or non-psychotic disorder cases. This heterogeneity arises mainly due to the differences in individual lifestyles, genetic characteristics, and poor socioeconomic and family conditions. 

Hence, to adjust for the overdispersion, a Poisson lognormal model was adopted, where the individual-level processes (leading to the variations in individual-level risks) were modeled using Poisson distribution; however, the intensity parameters of the model varied (within any neighborhood) following a Gamma (Γ) distribution. The resulting compound model has $Var\;\left[O_{ik}\right]>\lambda_{ik}$, where overdispersion can be captured and adjusted [40]. Following the work of Law et al. (2006), two Gaussian random-effects terms, $u_{ik}$ and $s_{ik}$, were included with Equation (5) to construct the targeted Poisson-Gamma model [40]. The inclusion of $u_{ik}$ and $s_{ik}$ helps capture the non-spatial and spatial structures in the unknown area-specific relative risks due to unmeasured or latent covariates. Thus, Equation (5) becomes

(6) $$\log\;[\lambda_{ik}]=\log\;[E_{ik}]+\beta_{0}+\beta_{1}X_{1i}+\beta_{2}X_{2i}+\beta_{3}X_{3i}+\beta_{4}X_{4i}+\beta_{5}X_{5i}+\beta_{6}X_{6i}+u_{ik}+s_{ik}$$

The models given by Equation (6) were fitted using the WinBUGS software (https://www.mrc-bsu.cam.ac.uk/software/bugs/the-bugs-project-winbugs/, accessed on 5 December 2019) from The BUGS Project (Cambridge, London). The prior information for the $\beta_{1}$, $\beta_{2}$, $\beta_{3}$, $\beta_{4}$, $\beta_{5},$ and $\beta_{6}$ terms were specified as a normal distribution with an expected mean of 0 and a precision (1/variance) of 0.00001. For the spatially non-structured $\left(u_{ik}\right)\;$and structured $\left(s_{ik}\right)$ random effect terms, an independent normal distribution and the intrinsic conditional autoregressive (ICAR) distribution were specified. The prior information of precision parameters for the unknown random effects was specified as a Γ distribution (a,b) with a mean of $\frac{a}{b}$ and variance of $\frac{a}{b^{2}}$. For this analysis, the prior distribution of Γ (0.001, 0.001) was used for both the random effect terms. The intercept term, $\beta_{0}$, was assigned with an improper uniform prior, dflat(), due to the inclusion of a sum-to-zero constraint on the random effects.

In order to understand the relative contributions of the spatially structured $\left(s_{ik}\right)$ and non-structured $\left(u_{ik}\right)\;$random effect terms, the posterior distribution of the quantity ψ was calculated, which could be expressed as follows [73]:

(7) $$\psi =\frac{SD_{s_{ik}}}{\left(SD_{s_{ik}}+SD_{u_{ik}}\right)}$$

where $SD_{s_{ik}}$ is the empirical marginal standard deviation of $s_{ik}$ and $SD_{u_{ik}}$ is the empirical marginal standard deviation of $u_{ik}$.

As ψ→1, the spatially structured random effect $\left(s_{ik}\right)$ would dominate the model compared to the non-structured effect $\left(u_{ik}\right)$; thus, the variation in the area-specific relative risk due to unmeasured covariates would be mainly spatial in nature. Conversely, when ψ→0, the non-structured random effect dominates the model, and the effect of spatial variation can be considered as negligible.

Initial values were assigned to the parameters, from which the estimation began and converged to the target posterior distribution. The convergence was checked by running two chains with widely differing initial values and by visual inspection of the trace plots, the serial autocorrelation function, and the Gelman-Rubin diagnostic. The trace plots were inspected to check whether the samples from the chains scattered around a stable mean, while the autocorrelation graphs were checked to see whether the graphs had approached zero. The Gelman-Rubin graphs were checked to observe whether the ratio of the between- and within-chain variances converged towards 1.0. 

Once convergence was reached, the accuracy of the posterior estimate was assessed using the Monte Carlo (MC) error of the posterior mean for each parameter. The accuracy of the estimation and the number of samples taken to generate the posterior estimate were considered satisfactory when the MC error was <5% of the sample (posterior) deviation. The deviance information criterion (DIC) and the number of effective parameters ($p_{D}$) for each model were recorded to assist the selection of the best model.

(8) $$DIC=\bar{D\;}+p_{D}\;$$

where $\bar{D}$ is the posterior mean of the deviance.

The model given by Equation (6) was repeated separately for psychotic and non-psychotic disorders and for each of the vegetation measures (EVI, NDVI, SAVI, Veg_RF, and Tree_OD). Hence, a total of 10 models were required for this part of the analyses. The models of the same outcome variable (for example, psychotic or non-psychotic) but using different vegetation measures were compared to understand the differential effect of vegetation measures on the association between vegetation and the two types of mental health disorders. In addition to DIC and $p_{D}$, comparisons between the models were made in terms of the area-specific relative risks and the role of different vegetation measures in determining the significance of the association. 

#### 2.2.8. Assessment of the Relative Risk of Mental Health Disorders Due to the Variations in Vegetation Content

The relative risk values from the models of the five vegetation measures were checked to understand if they substantially differed from one another. The posterior mean values from the Bayesian models and the median and the interquartile ranges of the relative risk values were assessed using box plot diagrams to analyze the differences in absolute magnitude. Afterward, the results from the Bayesian spatial models (95% CI, DIC and $p_{D}$) and the risk value assessments were used to select one (out of the five vegetation measures) to map the relative risks of psychotic and non-psychotic disorders in the study area. 

The relative risk being mapped was owing to the variations in vegetation content after adjusting for potential confounders and unmeasured covariates. Equations (3) and (6) show that the relative risk can be defined using the following model components:

(9) $$\begin{array}{cc} r_{ik}= & \exp\left[\beta_{0}\right]*\exp\left[\beta_{1}X_{1i}\right]*\exp\left[\beta_{2}X_{2i}\right]*\exp\left[\beta_{3}X_{3i}\right]*\exp\left[\beta_{4}X_{4i}\right]*\exp\left[\beta_{5}X_{5i}\right]\;*\exp\left[\beta_{6}X_{6i}\right]*\\ & \exp\;\left[u_{ik}\right]*\exp\;\left[s_{ik}\right]] \end{array}$$

## 3. Results

### 3.1. Vegetation Indices

**Figure 2:**
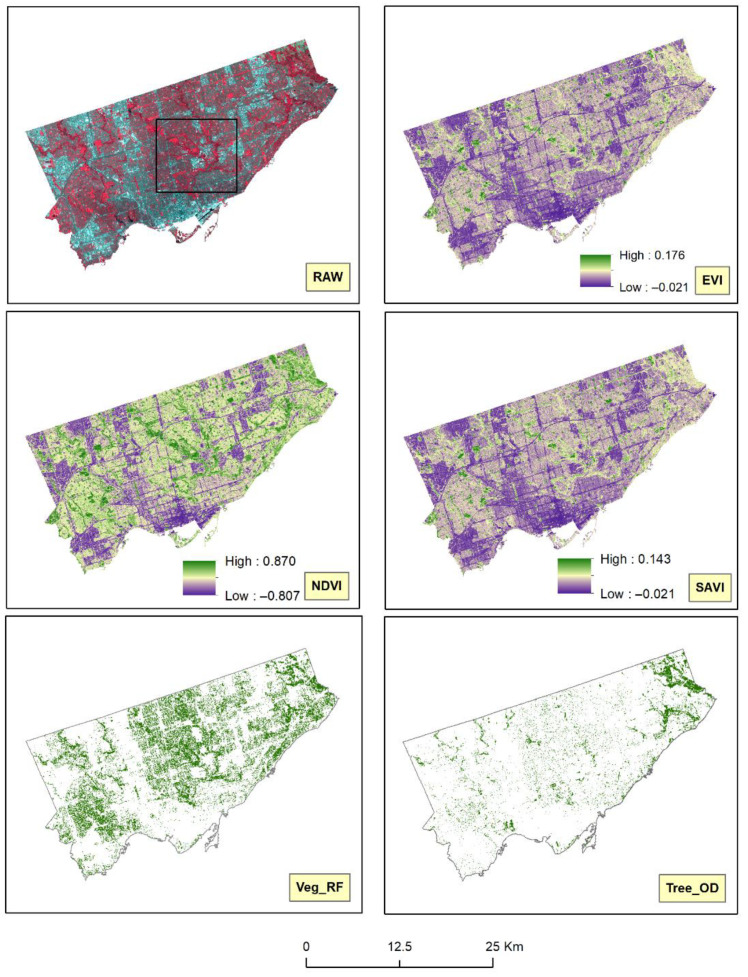
The macro-scale differences between the three vegetation indices and the area-based measures of vegetation cover. The shades of green represent the vegetation-covered areas for all the three vegetation indices, and the solid green color represents vegetation cover in the area-based measures. The black selection box in the raw image represents the portion of the study area that was zoomed-in in Figure 3 for better visualization of the micro-scale differences.

Figure 2 illustrates the false-color composite of the raw Landsat-8 image, the three vegetation indices (EVI, NDVI, and SAVI), and the area-based measures of vegetation cover (Veg_RF and Tree_OD). The false-color composite image displayed here utilizes the traditional color infrared image visualization technique for satellite images and the band combination of near-infrared, red and green (instead of red, green, and blue), to vibrantly illustrate vegetation in bright red color [14]. Accuracy assessments revealed quite high accuracies of the final LULC model used to derive the Veg_RF variable. The user’s accuracy and the Kappa coefficient values for the final LULC model were 0.967 and 0.909, respectively. The developed LULC model suggested that 22.5% of Toronto was covered by vegetation in 2016. 

**Figure 3:**
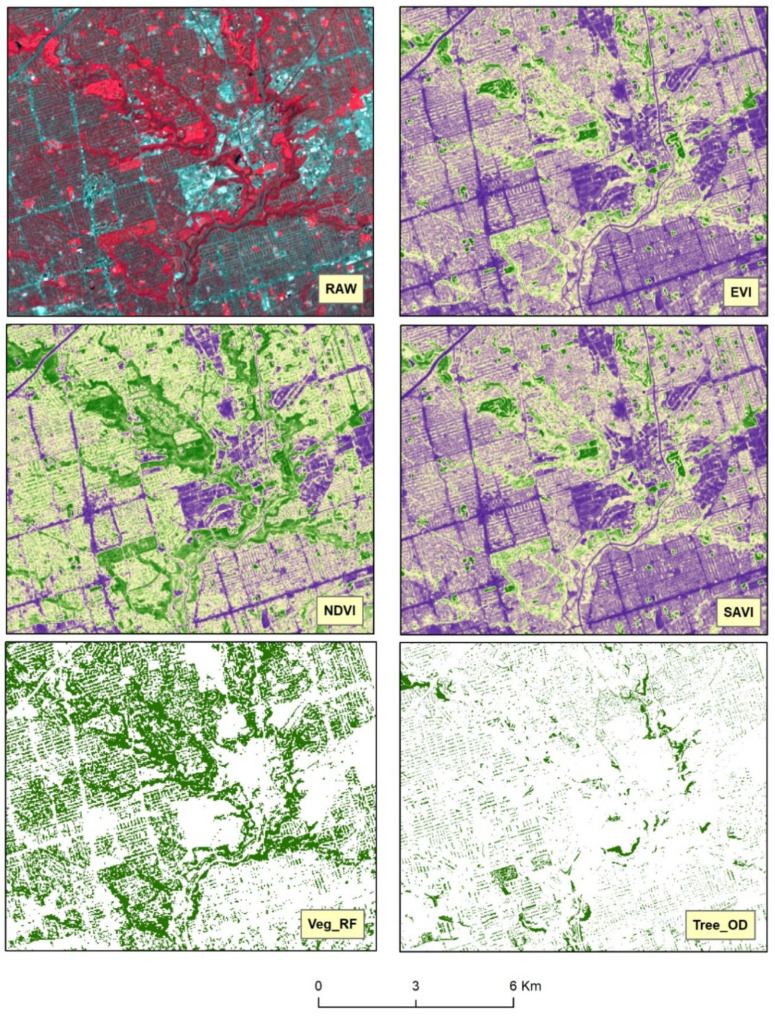
The micro-scale differences between the three vegetation indices and the area-based measures of vegetation cover. The shades of green represent the vegetation-covered areas for all the three vegetation indices, with darker shades of green representing dense and healthy vegetation. The yellow and the purple areas mainly represent the non-vegetation areas in the indices. The solid green color represents vegetation cover, while the white color represents non-vegetation regions in the area-based measures.

**Figure 4:**
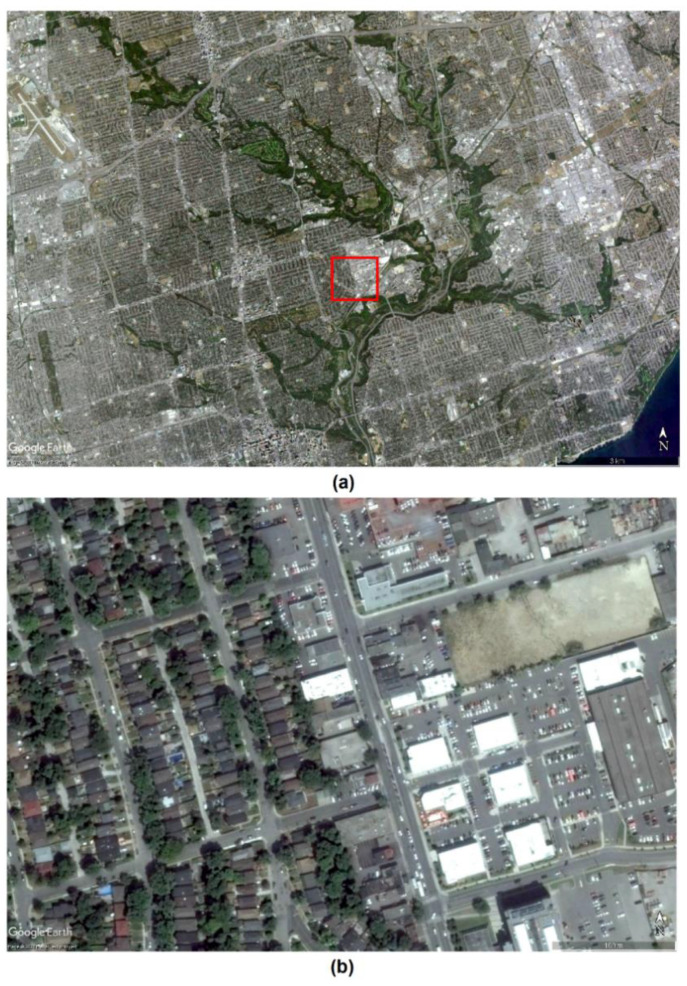
Google Earth images of a portion of the study area. The images show (**a**) a segment of the study area with vegetation cover and (**b**) a magnified image of the segment.

Comparing the different vegetation measures can provide further insights. Figure 2 and Figure 3 show that the three constructed vegetation indices showed a gradation of green color to illustrate both the density and health of the vegetation cover. Figure 3 also shows that the yellow patches in the NDVI contained a marked presence of green color compared to the other two indices. On closer inspection and further magnification of Figure 2, high-resolution Google Earth images in Figure 4 indicate that these yellow and small green patches actually represented built-up structures and surrounding vegetation, respectively. Hence, there is evidence of spectral confusion or false detection of other non-vegetation features as vegetation-covered areas.

Interestingly, despite having different computational processes (Supplementary File, Table S2), EVI and SAVI could be seen as more similar to each other compared to NDVI. In contrast, both area-based measures of vegetation only showed the areal-extent of vegetation, as indicated by the solid green color. The Tree_OD data severely underestimated the vegetation content compared to the other four satellite-derived vegetation measures. The areal extent of vegetation cover detected by Veg_RF matched more closely with NDVI as compared to EVI or SAVI. 

### 3.2. The Association between Vegetation and Psychotic and Non-Psychotic Disorders

The results of the Bayesian spatial modeling were used to analyze the association between various measures of vegetation and psychotic and non-psychotic disorders. The results of the analyses are tabulated in Table 4. The results indicate that only EVI and SAVI were significantly associated with both psychotic and non-psychotic disorders. These two vegetation indices were negatively associated with the number of psychotic and non-psychotic disorder cases, implying that low counts of mental health disorder cases were observed in areas with high EVI and SAVI values. The magnitude of the association between EVI and psychotic disorders was $\beta_{1}$ = −4.056 (95% CI: −8.147, −0.025) and that between EVI and non-psychotic disorders was $\beta_{1}$ = −2.442 (95% CI: −4.735, −0.172). Similarly, the magnitude of the association of SAVI with psychotic disorders was $\beta_{1}$ = −3.676 (95% CI: −7.350, −0.008) and that with non-psychotic disorders was $\beta_{1}$ = −2.213 (95% CI: −4.372, −0.121). Neither NDVI nor any of the area-based vegetation measures (Veg_RF and Tree_OD) showed any significant association with the psychotic and non-psychotic disorder cases. 

Among the confounding variables, ethnic concentration $\left(\beta_{3}\right)$, residential instability $\left(\beta_{4}\right),$ and the rate of substance use disorder $\left(\beta_{6}\right)$ showed statistically significant associations with both the psychotic and non-psychotic disorder cases. However, only ethnic concentration showed a negative association, implying that low counts of psychotic and non-psychotic disorder cases were observed in areas having high ethnic concentration. In contrast, material deprivation $\left(\beta_{2}\right)$ was found to be significantly and positively associated with psychotic disorders. The dependency $\left(\beta_{5}\right)$ variable did not exhibit any significant association with any of the two outcome variables. 

The values of ψ are greater than 0.50 in all the ten models and are all statistically significant. The ψ values for the models of psychotic disorders are close to 0.50; therefore, the spatially structured random effect term$\;\left(s_{ik}\right)$ and the non-structured random effect term $\left(u_{ik}\right)$ are almost equally dominant in the models. However, the values of ψ in the models for non-psychotic disorders are greater than 0.70 and are closer to 1 (ψ→1), showing that $s_{ik}$ dominated each of the models compared to $u_{ik}$. Therefore, the variations in the area-specific relative risk due to unmeasured covariates in the study area had notable spatial structures for both the psychotic and non-psychotic disorder cases. 

No discernible differences in the values of DIC and the number of effective parameters ($p_{D}$) are evident for the models analyzing the association between vegetation and psychotic disorders. Similar results were obtained for the models for non-psychotic disorders. These results demonstrated that for a specific outcome variable (for example, psychotic or non-psychotic disorders), using different vegetation measures did not affect the goodness of fit and the model parsimony. The most notable change observed from the results, therefore, is the difference in the significance of the association with the vegetation variables. The findings suggest that a significant association is detected only with the vegetation indices, specifically with the EVI and SAVI. 

**Figure 5:**
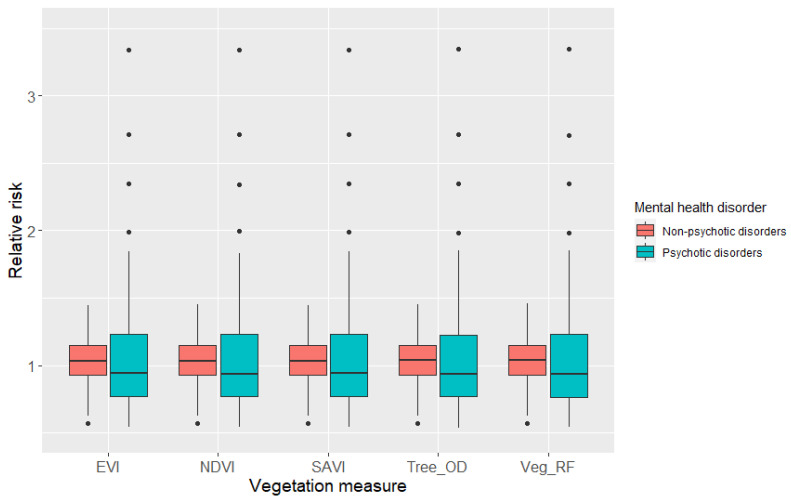
Box plot diagram showing the posterior mean of relative risks of psychotic and non-psychotic disorders. The relative risk values are shown for each of the five different vegetation measures in the 140 neighborhoods in Toronto.

The relative risk values $\left(r_{ik}\right)$ of psychotic and non-psychotic disorders, as defined by Equation (9), for each of the vegetation measures are shown in Figure 5. The median and the interquartile range of the box plots show that there are substantial differences in the relative risks for psychotic and non-psychotic disorders. However, the relative risk values are very similar for the five vegetation measures in both these mental health disorder categories. 

### 3.3. The Spatial Distribution of the Relative Risk of Psychotic and Non-Psychotic Disorders

**Figure 6:**
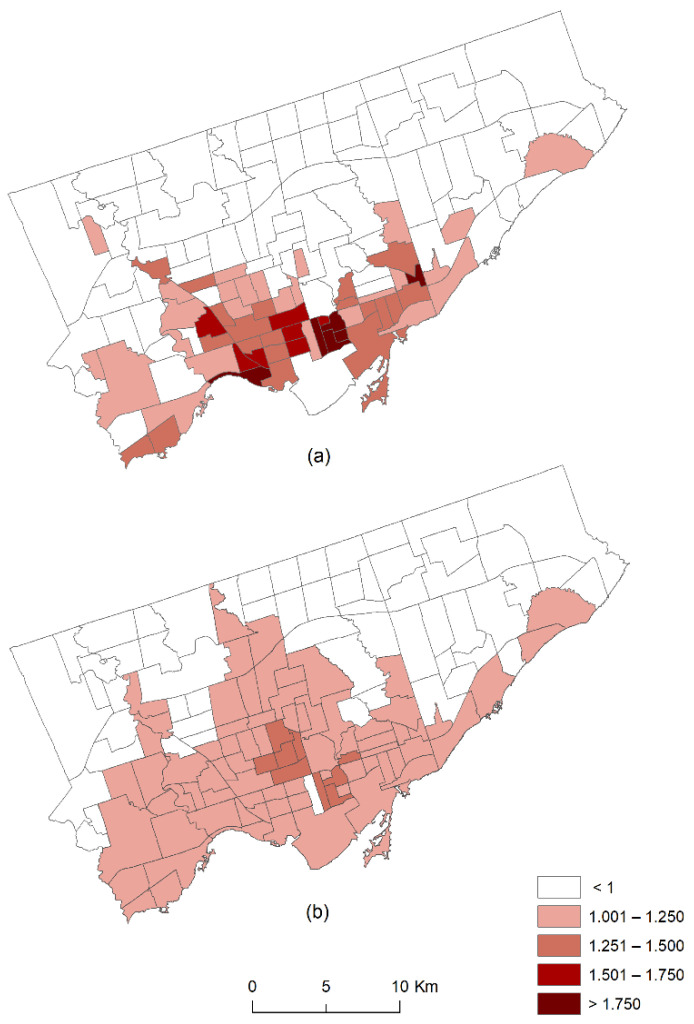
The posterior mean of the relative risk$\;\left(r_{ik}\right)\;$of (**a**) psychotic and (**b**) non-psychotic disorders.

Contrary to the non-spatial depiction of the relative risks using box plots, histograms and other different forms of charts, illustrating relative risks using maps can help accurately identify high-risk areas. The relative risk $\left(r_{ik}\right)$ from the EVI models for psychotic and non-psychotic disorders are shown in Figure 6a,b, respectively. The areas with relative risk values >1 could be interpreted as areas with high risk of psychotic or non-psychotic disorders due to reduced vegetation cover after adjusting for the risks from material deprivation, ethnic concentration, residential instability, dependency, substance use disorders, and the unmeasured covariates. 

Figure 6a shows that neighborhoods with the relative risk of psychotic disorders >1 were mostly clustered in the southern part and extended from the west to east. There were six neighborhoods with very high risk ($r_{ik}$ > 1.75) in the southcentral part of Toronto. In contrast, Figure 6b reveals that the neighborhoods with the relative risk of non-psychotic disorders >1 covered much of the southern and the northcentral parts of Toronto. When Figure 6a,b are considered together, it can be observed that the neighborhoods with high risk ($r_{ik}$ > 1) of psychotic disorders are also at high risk from non-psychotic disorders. However, unlike the relative risk of psychotic disorders, the relative risk of non-psychotic disorders did not exhibit very high values and was mostly below the value of 1.5. These two relative risk maps suggest that the northern part of Toronto is relatively at lower risk compared to the southern part.

## 4. Discussions

Based on the knowledge from available literature, this is the first study that employed Bayesian spatial statistics to analyze the performance of different vegetation measures in detecting a significant association between vegetation and mental health. This study provided empirical evidence that the type of vegetation measure in the model could influence the significance of the association. Furthermore, in this study, a significant association between vegetation and the psychotic and non-psychotic disorder cases was observed when EVI or SAVI was used as the vegetation measure. This suggests that the satellite-based vegetation indices, which are corrected for atmospheric disturbances, canopy background noise, and soil brightness, could help detect a significant association between vegetation and different types of mental health disorders. The observed results could be explained by the fact that these indices could provide detailed information on the quality of exposure to vegetation and, thus, people’s true exposure to surrounding greenness. The log-linear models (specifically the ψ values) revealed a strong dominance of the spatially structured unmeasured and latent covariates during the relative risk estimations. These latent covariates, if not adjusted in an epidemiological study, could potentially affect the detection of a significant association between vegetation and mental health and could also bias the risk estimation. 

This study found that the area-based measures of vegetation cover (Veg_RF and Tree_OD) were not associated with psychotic and non-psychotic disorders. This difference could be explained in terms of the differences in their functionality. Every day, people are regularly exposed to different forms of vegetation in their surroundings [1,74], which several studies have attempted to characterize using the term “surrounding greenness”. These studies found that both the density and health of vegetation are vital components for measuring the surrounding greenness in an area [75,76]. The extent to which vegetation cover can impart mental health benefits is directly dependent on the intensity and quality of the exposure to surrounding vegetation, which in turn depends on the richness of the vegetation cover and the duration of exposure [1,22]. In this regard, the area-based vegetation measures were simply based on the percentage of vegetation or tree cover in a neighborhood. Therefore, the values could not vary by the level of surrounding greenness to which people were exposed. Consequently, the association analyzed using these area-based measures could capture only a partial relationship between vegetation and mental health. 

Additionally, area-based measures that are based on the visual interpretation of aerial images can lead to inaccuracies in the detection of vegetation. In highly urbanized settings such as Toronto, with a marked presence of settlements that could reduce the visibility of trees and surrounding vegetation patches, such area-based measures of vegetation might not be suitable for health-related studies. Furthermore, the visual interpretation process is also subject to the interpretation of the user or the ability to identify different types of vegetation in the image. Consequently, this type of dataset might underestimate the vegetation content in the area, as evidenced when visually comparing the raster images of satellite-based vegetation measures (EVI, NDVI, SAVI, and Veg_RF) with the area-based measure of tree cover (Tree_OD).

Although the visual interpretation process could be automated through the application of powerful machine learning ensembles such as random forest classifiers, a high degree of landscape heterogeneity, such as that present in an urban setting, could preclude the accurate detection of different types of vegetation in the area [55,77]. In addition, medium to low resolution of Landsat images (30 m) may lead to spectral confusion and problems in differentiating vegetation from other land cover classes [55,77]. This misclassification of vegetation may lead to the increased risk of misinterpreting the actual relationship between vegetation and mental health disorders. However, the accuracy assessments revealed that the RF model in this study had over 90% accuracy for the land cover classification model, so over- and under-estimation should not be a problem for this study. Thus, the inability of Veg_RF to capture the density and biomass conditions of vegetation cover or people’s actual exposure to surrounding vegetation could be the reason for the differences observed in the results of Bayesian models using Veg_RF and the vegetation indices (EVI and SAVI). 

Contrary to the area-based measures, satellite-based vegetation indices such as EVI, NDVI, and SAVI can measure both the density and quality of the vegetation cover. This is because their values vary based on the chlorophyll content, variations in canopy cover, and canopy architectures [25,26,28]. For example, the values of the vegetation indices increase when there are more leaves and more photosynthetic activities in the vegetation patch, which are the measures of density (leaves) and greenness, respectively. Therefore, using these indices can help accurately capture the relationship between the surrounding greenness and poor mental health outcomes [1], as the number of mental health disorder cases is allowed to vary by both the density and health of the surrounding vegetation cover. This could have led to the differences in the results of Bayesian models from the vegetation indices (EVI and SAVI) and area-based measures (Veg_RF and Tree_OD). 

Surprisingly, the models for NDVI did not yield any statistically significant association with any of the psychotic or non-psychotic disorders. This could be explained in terms of the computational differences between NDVI and the other two indices. First, NDVI and SAVI are computationally similar, but SAVI could be considered as a modified form of NDVI, where the NDVI is corrected for the influence of soil brightness [49]. The background brightness from surfaces such as soil may interact with the radiation reflected towards the sensor (satellite) from the overlying vegetation canopy [25] and may result in complex soil surface–vegetation interactions that might affect the values of NDVI. Thus, in a highly urbanized area such as Toronto, substantial background noise from the different built-up surfaces such as bitumen covered roads, concrete pavement, brick surfaces, and gravel-covered rooftops (see Figure 4b) could impair NDVI’s vegetation detection capacity. Second, the natural atmospheric conditions in urban areas are frequently disrupted by pollutants from vehicles and commercial sites [78]. Additionally, urban morphology such as tall buildings and surface roughness as well as the low heat capacity of materials such as concrete can affect the wind flow and both vertical and horizontal distributions of these pollutants in the atmosphere [79]. These atmospheric disturbances can affect the transmittance of the red band through the atmosphere to the satellite and thus can influence the NDVI or SAVI values. The EVI can overcome this problem and can adjust for the atmospheric disturbances by using the atmosphere-sensitive blue band to correct the affected red band for atmospheric influences [80]. EVI is also adjusted for canopy background noise through the canopy signal decoupling process, which makes it very sensitive to vegetation greenness. The decoupling process allows different forms of vegetation to be captured by minimizing the covering effect of large, overlying vegetation [24,80,81]. Therefore compared to relatively simpler indices such as NDVI or SAVI, EVI could be better at capturing people’s exposure to the different types of vegetation cover in their surroundings. 

Although this study could not find any notable differences between the vegetation cover detected by EVI and SAVI, the atmospheric perturbations and the canopy background noise could cause a substantial difference between these two indices in other urban areas. Therefore, it is highly recommended to consider the type of study area (urban, peri-urban, or rural) before selecting vegetation indices for mental health studies. Considering the urban geophysical settings and the potential atmospheric and environmental disturbances that could be present in Toronto, we preferred EVI over SAVI to map the relative risks. 

The results of this study showed that the vegetation was negatively associated with psychotic and non-psychotic disorders in Toronto, after adjusting for potential confounders and unmeasured covariates. Comparing the results from the models having the same vegetation measure but different mental health outcomes, it could be found that areas with greater vegetation cover had fewer counts of psychotic disorder cases compared to non-psychotic disorders. A possible explanation of the observed relationship could be that vegetation was protective against the onset of mental health disorders, or people living with mental health disorders tend to reside in areas with poorer vegetation. Future studies could further investigate this. 

In this regard, the mental health benefits from vegetation can be broadly categorized into two specific domains: reducing harm and improving restoration capacities [1]. Vegetation can help reduce physical harm to the body by improving environmental conditions, such as by reducing air pollution and exposure to heat and noise. These factors adversely affect the psychological well-being and cognitive development of people, which could later transform into mental health disorders [22,82,83]. Regular exposure to greenery and the natural environment can help improve stress response and circumvent negative emotions that deteriorate mental health conditions. In this process, attention restorative capacities improve as well, as people have better cognition, which helps willfully direct attention to the positive aspects of life [1,84]. 

This research has highlighted several critical issues of studying the relationship between vegetation and mental health disorders. First, this study established the necessity to carefully select a vegetation measure for accurately capturing the quality of people’s exposure to surrounding greenness. Second, this study showed that the physical settings of an area could introduce problems while detecting surrounding vegetation and thus can affect the significance of the association between vegetation and mental health. Third, the negative association between vegetation content and the number of psychotic and non-psychotic disorder cases reflects that investments in urban vegetation can have tangible health benefit effects, such as improved mental health conditions of the general public. This research has provided directions that could be extended further to design future studies to understand how long-term investments in urban vegetation could help reduce healthcare costs. 

Fourth, while this study did not measure the therapeutic impact of vegetation on recovery from mental disorders, the evidence shows that mental disorders are less common in higher-quality vegetation areas. Future research should incorporate longitudinal studies to explore the impact of constant exposure to vegetation cover on the incidence and treatment of psychotic and non-psychotic disorders. Finally, the analyses conducted in this study quantified the relative contributions of the spatial and non-spatial latent covariates and showed that these covariates could significantly influence the prevalence of mental health disorder cases. The findings also showed that latent risk factors have considerable spatial structure; thus, any interventions targeting the control of mental health disorder cases should adopt spatial approaches. 

The findings of this study may have direct implications in meeting the Sustainable Development Goals (SDGs) [85]. By 2030, the SDG Target 3.4 aims at a one-third reduction in premature mortality from non-communicable diseases through the prevention, treatment, and promotion of mental health and well-being [86]. The SDG Target 11.7 aims at providing universal access to safe and inclusive green and public spaces, particularly to people living in cities and urban areas [87]. Therefore, if intervention strategies are undertaken based on the findings of this study that reduced vegetation cover is significantly associated with the two prevalent mental health disorders in urban areas, it can help meet the SDG Targets 3.4 and 11.7. Our findings suggest that the mental health and well-being of the urban population could be improved through increased access to public spaces with abundant vegetation cover. 

Despite its strengths, several limitations are present in this study. First, research suggests that the surrounding greenness and exposure to greenness are best captured by the eye-level panoramic imagery of green space [1]. However, the process of obtaining such imagery is both time-consuming and expensive. Therefore, this study attempted to demonstrate the performance of the vegetation measures using datasets that are inexpensive and readily available for epidemiological research. Second, this is an ecological study, where the results conform to the findings relevant at the area level for groups of people. The results of the associations need to be interpreted with caution, and no individual-level conclusions should be drawn from the findings. Additionally, the study was carried out for a single city in Canada with specific geophysical and socioeconomic settings. Therefore, the results should not be generalized, especially for areas with contrasting geophysical and socioeconomic conditions. Third, the study did not assess the performance of an index that utilizes the combined strengths of both EVI and SAVI. Unfortunately, such an attempt is well beyond the scope of this study, as it requires a careful selection of techniques to combine the two indices or perform the adjustments that are conducted during the calculation of these indices. Finally, we were not able to analyze the impact of vegetation within the different mental health disorder categories in our psychotic and non-psychotic disorder datasets. Future studies could focus on studying the impact of vegetation on these categories, since it is likely that vegetation may have differential impacts on different mental health conditions.

Regardless of these limitations, this study has taken up the challenge to identify the methodological constraints owing to the selection of different vegetation measures in population-based mental health studies. This research attempted to understand the complex relationship between vegetation and mental health disorders by developing hierarchical models that adjust for potential confounders and unmeasured covariates.

## 5. Conclusions

The increase in global urbanization and the subsequent loss of vegetation-covered areas are likely to put millions of people at risk from poor mental health conditions. Unfortunately, due to the disagreements between carefully designed studies, it is still unclear whether reduced vegetation is a significant risk factor for mental health disorders. In epidemiological research, a considerable challenge exists when selecting an appropriate vegetation measure to capture the different forms of vegetation in urban areas. Hence, there is a need to assess the performance of different types of vegetation measures in studying the association between vegetation and mental health disorders. Therefore, through the application of remote sensing, geographic information systems, and machine learning techniques, three satellite-based indices and two area-based measures of vegetation were used to analyze the relationship between vegetation and psychotic and non-psychotic disorders, after adjusting for material deprivation, ethnic concentration, residential instability, dependence, the rate of substance use disorders, and unmeasured (latent) covariates. The results from this analysis were further investigated to select a suitable vegetation measure, which was later employed to map the relative risk of psychotic and non-psychotic disorder cases in the study area. The associations were studied using Poisson-lognormal models under a Bayesian framework. The vegetation was found to be negatively associated with both psychotic and non-psychotic disorder cases. Results suggest that satellite-based indices could be better than area-based measures at capturing a significant association with mental health. The findings also indicate that the indices, such as enhanced vegetation index and soil adjusted vegetation index, which are adjusted for atmospheric disturbances, canopy background, and soil-brightness, could be particularly useful, especially in an urban context. The relative risk maps provided evidence of spatial variations in risk from psychotic and non-psychotic disorders, which could be the focus of targeted public health interventions. The findings from this study are expected to provide critical guidelines on the selection of an appropriate vegetation measure for future population-based mental health studies. The findings could also be helpful for other health research that uses such measures to understand the exposure of the general public to surrounding vegetation cover. Our study suggests that policymakers should prioritize the issue of decreased greenness in densely populated areas for the development of public health policy initiatives that aim to mitigate any adverse impact of vegetation loss on the mental well-being of urban residents.

## Figures and Tables

**Table 1:** Categories and sub-categories of mental health disorders used in this study.

Type	Sub-Category	OHIP Codes of Sub-Category
Psychotic disorders	Schizophrenia	295
	Manic-depressive psychoses, involutional melancholia	296
	Other paranoid states	297
	Other psychoses	298
Non-psychotic disorders	Anxiety neurosis, hysteria, neurasthenia, obsessive-compulsive neurosis, reactive depression	300
	Personality disorders	301
	Sexual deviations	302
	Psychosomatic illness	306
	Adjustment reaction	309
	Depressive disorder	311

**Table 2:** Land cover classes developed in this study.

LULC Types	Description
Bare soil	Exposed soils, construction sites
Built-up	Residential, commercial and services, industrial, transportation, roads, mixed urban, and other urban
Vegetation	Deciduous forest, mixed forest lands, palms, conifer, scrub, and others
Waterbody	Permanent and seasonal wetlands, inland water bodies, low-lying areas, marshy land, rills and gully, swamps

**Table 3:** Summary statistics of the key variables used to study the association between vegetation and mental health disorders.

Variables	Minimum	Mean (Standard Deviation)	Maximum
**Dependent Variables**			
Number of psychotic disorders	94	282.864 (±152.637)	861
Number of non-psychotic disorders	757	2239.850 (±964.286)	5523
**Independent Variables (vegetation)**			
EVI	0.037	0.052 (±0.006)	0.0679
NDVI	0.473	0.561 (±0.035)	0.634
SAVI	0.041	0.058 (±0.006)	0.075
Percentage of vegetation cover (Veg_RF)	0.501	20.730 (±13.267)	54.279
Percentage of tree cover (Tree_OD)	0.100	6.540 (±5.611)	34.117
**Independent Variables (others)**			
Material deprivation (OMI)	−1.520	0.250 (±0.895)	3.068
Residential instability (OMI)	−0.785	0.723 (±0.783)	3.009
Dependency (OMI)	−1.262	−0.228 (±0.393)	0.897
Ethnic concentration (OMI)	−0.317	0.902 (±0.838)	3.282
Substance use disorder rate	2.410	9.988 (±4.392)	30.54

**Table 4:** Summaries of results from Bayesian spatial modeling to analyze the association between vegetation and psychotic and non-psychotic disorders. The italicized values are significant at a 95% credible interval (CI).

Posterior Means Summaries	EVI	NDVI	SAVI	Veg_RF	Tree_OD
**Psychotic disorders**					
$\beta_{0}$ (95% CI)	*−0.287* *(−0.514, −0.057)*	−0.148 (−0.508, 0.206)	*−0.286* *(−0.513, −0.059)*	*−0.477* *(−0.583, −0.375)*	*−0.492* *(−0.591, −0.395)*
$\beta_{1}$ (vegetation measure) (95% CI)	*−4.056* *(−8.147, −0.025)*	−0.626 (−1.249, 0.000)	*−3.676* *(−7.350, −0.008)*	−0.001 (−0.005, 0.004)	−0.001 (−0.006, 0.005)
$\beta_{2}$ (material deprivation) (95% CI)	*0.122* *(0.077, 0.166)*	*0.117* *(0.073, 0.161)*	*0.121* *(0.076, 0.165)*	*0.108* *(0.062, 0.153)*	*0.112* *(0.068, 0.156)*
$\beta_{3}$ (ethnic concentration) (95% CI)	*−0.118* *(−0.169, −0.064)*	*−0.118* *(−0.169, −0.065)*	*−0.117* *(−0.169, −0.063)*	*−0.121* *(−0.172, −0.067)*	*−0.123* *(−0.175, −0.067)*
$\beta_{4}$ (residential instability) (95% CI)	*0.179* *(0.135, 0.221)*	*0.180* *(0.137, 0.221)*	*0.179* *(0.135, 0.221)*	*0.179* *(0.136, 0.221)*	*0.181* *(0.138, 0.223)*
$\beta_{5}$ (dependency) (95% CI)	−0.057 (−0.124, 0.011)	−0.057 (−0.125, 0.011)	−0.056 (−0.124, 0.012)	−0.057 (−0.126, 0.012)	−0.061 (−0.130, 0.008)
$\beta_{6}$ (substance use disorder) (95% CI)	*0.041* *(0.033, 0.049)*	*0.041* *(0.033, 0.049)*	*0.041* *(0.033, 0.049)*	*0.041* *(0.033, 0.049)*	*0.041* *(0.033, 0.049)*
ψ (95% CI)	*0.537* *(0.231, 0.792)*	*0.519* *(0.223, 0.779)*	*0.539* *(0.236, 0.792)*	*0.501* *(0.203, 0.787)*	*0.522* *(0.213, 0.798)*
$p_{D}$	102.66	102.589	102.642	103.662	103.683
**DIC**	1271.530	1271.580	1271.560	1272.160	1272.110
**Non-psychotic disorders**					
$\beta_{0}$ (95% CI)	0.098 (−0.031, 0.230)	0.015 (−0.195, 0.227)	0.098 (−0.037, 0.236)	*−0.073* *(−0.135, −0.012)*	*−0.062* *(−0.122, −0.003)*
$\beta_{1}$ (vegetation measure) (95% CI)	*−2.442* *(−4.735, −0.172)*	−0.081 (−0.446, 0.280)	*−2.213* *(−4.372, −0.121)*	0.002 (−0.002, 0.006)	0.004 (−0.001, 0.008)
$\beta_{2}$ (material deprivation) (95% CI)	0.014 (−0.014, 0.041)	0.009 (−0.019, 0.036)	0.013 (−0.015, 0.040)	0.015 (−0.012, 0.041)	0.007 (−0.020, 0.033)
$\beta_{3}$ (ethnic concentration) (95% CI)	*−0.114* *(−0.147, −0.082)*	*−0.115* *(−0.148, −0.082)*	*−0.114* *(−0.146, −0.081)*	*−0.115* *(−0.147, −0.083)*	*−0.107* *(−0.140, −0.075)*
$\beta_{4}$ (residential instability) (95% CI)	*0.055* *(0.028, 0.082)*	*0.057* *(0.029, 0.084)*	*0.055* *(0.028, 0.082)*	*0.062* *(0.035, 0.089)*	*0.056* *(0.029, 0.082)*
$\beta_{5}$ (dependency) (95% CI)	0.007 (−0.032, 0.046)	0.006 (−0.034, 0.045)	0.007 (−0.031, 0.046)	−0.002 (−0.041, 0.037)	0.007 (−0.031, 0.046)
$\beta_{6}$ (substance use disorder) (95% CI)	*0.011* *(0.005, 0.017)*	*0.011* *(0.005, 0.017)*	*0.011* *(0.005, 0.017)*	*0.011* *(0.005, 0.017)*	*0.011* *(0.006, 0.017)*
ψ (95% CI)	*0.750* *(0.595, 0.863)*	*0.754* *(0.595, 0.867)*	*0.750* *(0.596, 0.863)*	*0.744* *(0.593, 0.860)*	*0.755* *(0.601, 0.866)*
$p_{D}$	126.554	127.088	126.678	125.982	126.780
**DIC**	1591.070	1591.540	1591.290	1590.810	1590.750

ψ= relative contribution of the spatially structured and non-structured random effect terms; $p_{D}=$ number of effective parameters; DIC = deviance information criterion.

## Data Availability

Data is available in a publicly accessible repository that does not issue DOIs. Publicly available datasets were analyzed in this study. This data can be found here:
(1) Ontario Community Health Profiles Partnership: http://www.ontariohealthprofiles.ca/dataTablesON.php?varTab=HPDtbl&select1=7 (2) Ontario Primary Care Need, Service Use, Providers and Teams, and Gaps in Care 2015/16: http://www.ontariohealthprofiles.ca/dataTablesICES.php?varTab=HPDtbl&select1=7 (3) USGS-EarthExplorer: https://earthexplorer.usgs.gov/ (4) Open Data—Toronto (About Topographic Mapping—Treed Area): https://open.toronto.ca/dataset/topographic-mapping-treed-area/

## Supplementary Materials

The following are available online at https://www.mdpi.com/article/10.3390/ijerph18094713/s1; Table S1. Results of the test for detecting spatial autocorrelation in the data (global Moran's I test); Table S2. Details of the vegetation indices used in this study; Table S3. The four major dimensions of OMI with their indicators; Table S4. The result of the Pearson correlation coefficient test on the four OMI variables; Figure S1. Showing the interrelationships amongst the four OMI variables; Table S5. The models used to generate the multicollinearity test statistics.
