# Supplementary material for: Understanding the Differential Impact of Vegetation Measures on Modeling the Association between Vegetation and Psychotic and Non-Psychotic Disorders in Toronto, Canada
Source: Int J Environ Res Public Health. 2021 Apr 28;18(9):4713. doi: 10.3390/ijerph18094713 (PMC8124936; doi:10.3390/ijerph18094713)
Supplement: Supplementary file 1 [file ijerph-18-04713-s001.zip › Supplementary File (S1).pdf]

A) Details of the mental health disorder dataset

Enrollment, Access, Continuity and Mental Health Gaps in Care [1]

Dataset: Prevalence of mental health disorders and substance use by age, sex, and enrolled/non-enrolled status in City of Toronto and LHIN 7 by neighbourhood, 2015/16

ICES Project No.: 2018 0900 992 000

Data sources: A number of data sources were used to prepare this dataset. The sources and the type of data extracted are listed below:

a) ***OHIP - Ontario Health Insurance Plan:*** Health care provider claims
b) ***RPDB - Registered Persons Database:*** Ontario population and OHIP eligibility data
c) ***CPDB - Corporate Provider Database:*** Physician and group data from the Ministry of Health
d) ***IPDB - ICES Physician Database:*** Annual physician demographics, specialization and workload
e) ***CONTACT:*** Yearly health services contact and RPDB eligibility summaries
f) ***CAPE - Client Agency Program Enrollment:*** Registry of patients enrolled in primary care groups
g) ***CIC - Immigration, Refugees and Citizenship Canada (IRCC)'s Permanent Resident Database:*** Ontario portion of IRCC's Permanent Resident Database, including immigration application records for people who initially applied to land in Ontario

Study period: Fiscal 2015 (April 1, 2015 to March 31, 2016)

Study population: All Ontario permanent residents who are eligible for coverage under the publically-funded Ontario Health Insurance Plan (OHIP) on March 31, 2016

Inclusion / Exclusion Criteria:

a) **Inclusion criteria:**
All Ontario permanent residents eligible for OHIP coverage on March 31, 2016.

b) **Exclusion criteria:**
1. Invalid IKN
2. Death before March 31st, 2016
3. No contact within 8 years prior to March 31, 2016
4. Age>105 years
5. People living in long-term care and complex continuing care during the study period

Indicators: Mental health disorders were measured using outpatient visit/claim (OHIP)

Numerator: The number of individuals who had OHIP claims for the mental health conditions listed in the 'Data preparation' section in Table 1 of the paper.

Denominator: Total number of people who had a valid health card number and were alive on March 31, 2016

B) Testing for the spatial autocorrelation in mental health data using global Moran's I analysis

The global Moran's I test was executed on the age and sex-standardized rates (per 1000 population) of both sexes for psychotic and non-psychotic disorders. This analysis helped to understand whether there is a statistically significant spatial autocorrelation in the data. Based on the results of this test, the modeling technique for studying the association was selected.

The test was repeated for each of the psychotic and non-psychotic disorder data and the first-order Queen's case contiguity was used to define the spatial weight matrix. This weight matrix helped to identify the adjacent neighbors of each neighborhood in the Toronto area and evaluated the similarity and dissimilarity between the values of each neighborhood and its corresponding neighbors. The global Moran's I values range from -1 to + 1, where a highly negative value (Moran's I → -1) will correspond to a perfect dispersion of the mental health disorder cases and a value of 0 will correspond to a random distribution. In contrast, a highly positive value (Moran's I → +1) will indicate a marked spatial autocorrelation in the data and that the like values (high or low) are highly clustered together. The pseudo-p-values, which assessed the significance of the Moran's I values, were generated using 999 permutations. The results of the tests are summarized in Table S1.

Table S2. Results of the test for detecting spatial autocorrelation in the data (global Moran's I test).

Type	Moran's I value	p-value	Pattern
Psychotic disorder	0.508	0.001	Moderately clustered
Non-psychotic disorder	0.770	0.001	Highly clustered

Table S1 shows that the psychotic and non-psychotic disorder cases show a moderate and high clustering, respectively. A Moran's I value close to 0.5 and 0.7 was considered moderately and highly clustered, respectively. These results confirmed the need to use a spatial modeling technique that adjusts for spatial autocorrelation in the data.

C) Formulas used to calculate the vegetation indices

Table S2. Details of the vegetation indices used in this study.

Vegetation Indices	Formula	Description
**Enhanced Vegetation Index (EVI)**	**Generic:**<br>EVI = G * ((NIR - R) / (NIR + C1 * R – C2 * B + L_{EVI}))<br>**For Landsat 8:**<br>EVI = 2.5 * ((Band 5 – Band 4) / (Band 5 + 6 * Band 4 – 7.5 * Band 2 + 1))	EVI is a vegetation index that quantifies the vegetation greenness. Compared to other similar indices, EVI adjusts atmospheric conditions and canopy background noise and is more sensitive in areas with dense vegetation [2]<br><br>The higher the value of EVI, the greater is the vegetation content and the greenness of the area.
**Normalized Difference Vegetation Index (NDVI)**	**Generic:**<br>NDVI= (NIR - R) / (NIR + R)<br><br>**For Landsat 8:**<br>NDVI = (Band 5 – Band 4) / (Band 5 + Band 4)	NDVI is the most commonly used vegetation index in health research and can help estimate the greenness or the quality of vegetation cover [3].<br><br>However, in contrast to EVI, NDVI cannot adjust for the atmospheric conditions and canopy background noise.
**Soil Adjusted Vegetation Index (SAVI)**	**Generic:**<br>SAVI= ((NIR - R) / (NIR + R + L_{SAVI})) * (1 + L_{SAVI})<br><br>**For Landsat 8:**<br>SAVI = ((Band 5 – Band 4) / (Band 5 + Band 4 + 0.5)) * (1.5)	Although SAVI is similar to NDVI, it can adjust for the influence of the soil brightness, which otherwise affects the estimation of NDVI in areas where the vegetation cover is low [4].
**Band descriptions**		
NIR is the Near Infrared band of the satellite image<br>R is the Red band of the satellite image<br>B is the Blue band of the satellite image<br><br>G is the gain factor that makes EVI comparable to that of other vegetation indices such as NDVI<br>L_{EVI} is a constant used to adjust for the canopy background<br>L_{SAVI} is the soil brightness correction factor<br>C1 and C2 are constants used to adjust for atmospheric resistance		

D) Details of the socioeconomic covariates retrieved from the Ontario Marginalization Index (OMI)

The OMI comprises of four major dimensions or categories, these are:

1) Material Deprivation: This dimension was created from the indicators that measure income, quality of housing, education attainment and family structure characteristics such as family who are lone-parent families. Material deprivation is directly related to poverty and people's capacity to access and avail basic necessities.

2) Residential Instability: This dimension was constructed from the indicators that measure the types and density of residential accommodations and certain family structure characteristics such as the proportion of the population who are single, divorced or widowed. Residential instability captures the quality of neighborhoods, cohesiveness and supports in terms of these indicators.

3) Dependency: This dimension originated from the indicators that measure the area-level concentrations of people who are not compensated for their work or who do not receive income from employment. This group comprises of seniors, children, and people with disabilities.

4) Ethnic concentration: This dimension was made from the indicators that measure high area-level concentrations of people who are recent immigrants and people who belong to a visible minority group

A detailed list of the indicators used to create each of the four variables could be found in the Table S3.

Table S3. The four major dimensions of OMI with their indicators.

Material Deprivation	Ethnic Concentration	Residential Instability	Dependency
Proportion of the population aged 20+ without a high-school diploma	Proportion of the population who are recent immigrants (arrived in the past 5 years)	Proportion of the population living alone	Proportion of the population who are aged 65 and older
Proportion of families who are lone parent families	Proportion of the population who self-identify as a visible minority	Proportion of the population who are not youth (age 5-15)	Dependency ratio (total population 0-14 and 65+ / total population 15 to 64)
Proportion of total income from government transfer payments for population aged 15+		Average number of persons per dwelling	Proportion of the population not participating in labour force (aged 15+)
Proportion of the population aged 15+ who are unemployed		Proportion of dwellings that are apartment buildings	
Proportion of the population considered low-income		Proportion of the population who are single/divorced/widowed	
Proportion of households living in dwellings that are in need of major repair		Proportion of dwellings that are not owned	
		Proportion of the population who moved during the past 5 years	

E) Pearson correlation coefficient and multicollinearity tests of the Ontario Marginalization Index (OMI) variables

Prior to running the Pearson correlation coefficient and multicollinearity tests, the individual relationships amongst the four OMI dimensions were assessed using graphical representations. Figure S1 shows the inter-relationships amongst the OMI variables. For the most part, the graphs indicate that there is no notable linear association between the variables.

1) Pearson correlation coefficient test

The Pearson correlation coefficient test was used to assess the linear association between two or more OMI variables. Highly positive values correspond to positive associations between the tested variables and highly negative values correspond to negative associations. The correlation coefficient values range from -1 to +1. In general, the correlation coefficients having absolute values:

a) 0 to 0.25 represent a low correlation
b) 0.25 to 0.50 represent a moderately low correlation
c) 0.50 to 0.75 represent a moderate correlation
d) 0.75 to 1 represent a high correlation

The results tabulated in Table S4 indicate that except for correlations between instability and dependency, and between deprivation and ethnic concentration, the magnitudes of the remaining correlations were very small. The correlations coefficient value for instability and dependency was moderately low (> 0.5). The correlation coefficient for deprivation and ethnic concentration indicates a moderate correlation (> 0.75).

Table S4. The result of the Pearson correlation coefficient test on the four OMI variables.

Variables	Instability	Deprivation	Dependency	Ethnic concentration
Instability	**1***	**-0.200****	**-0.458***	-0.056
Deprivation		**1***	0.101	**0.649***
Dependency			**1***	**0.175****
Ethnic concentration				**1**

** significant at $p < 0.01$*
***significant at $p < 0.05$*

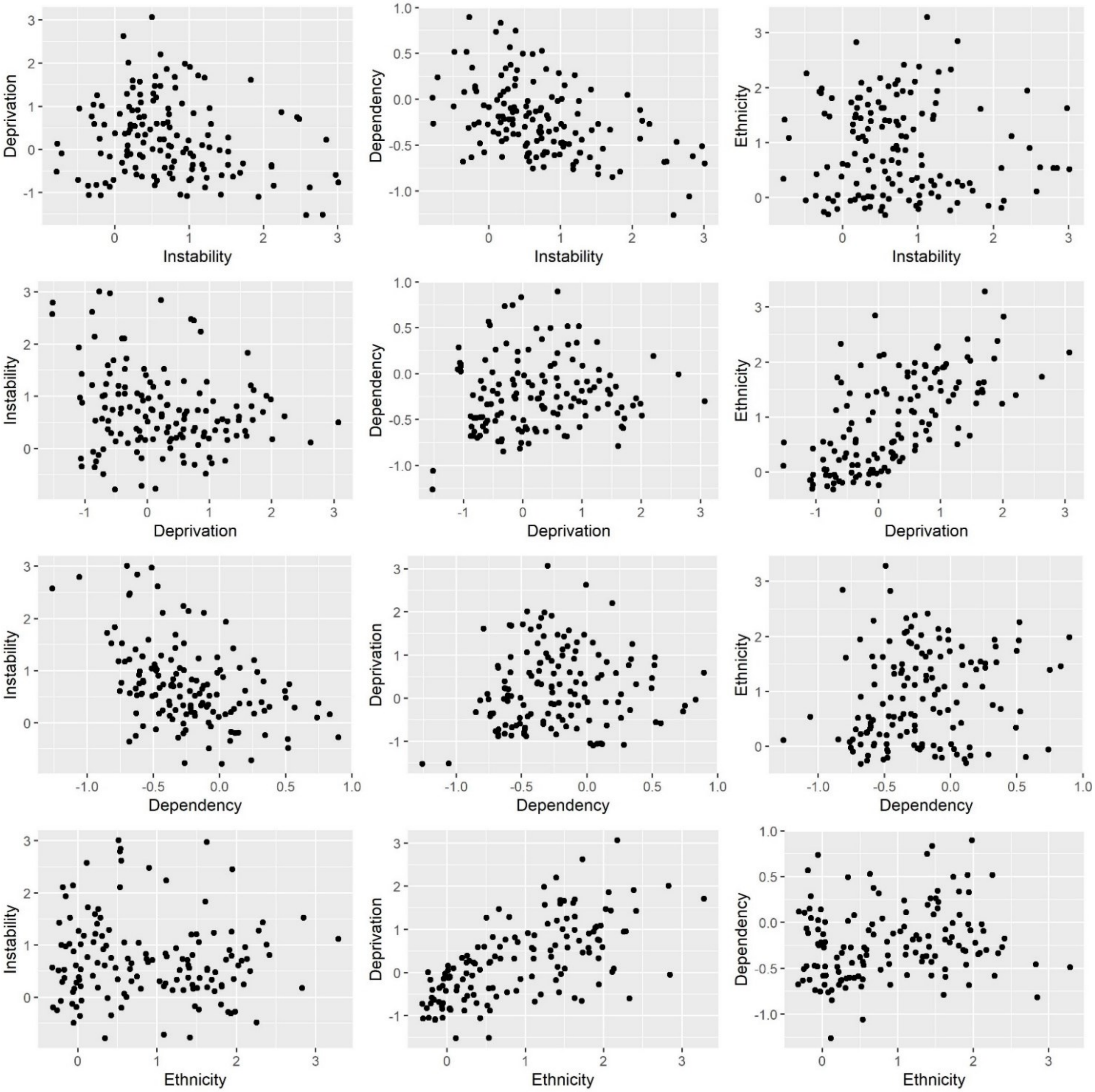

Figure S1. Showing the interrelationships amongst the four OMI variables.

2) *Multicollinearity test:*

As the Pearson correlation coefficient test could only evaluate the linear relationship between two variables at a single time, a multicollinearity test was conducted using the *olsrr* package in R (https://cran.r-project.org/web/packages/olsrr/olsrr.pdf). Multicollinearity occurs when the predictor or independent variables in a regression model are strongly linearly correlated with each other or show high inter-associations [5].

For this study, the Tolerance and the Variance Inflation Factors (VIF) were assessed to understand the extent of multicollinearity amongst the variables.

The details of the test statistics and their results are provided below:

a) Tolerance (1- R^2):

The tolerance is calculated by regressing one of the four OMI variables (the kth predictor) on rest of the three OMI variables. The R^2 value (R_k^2) is computed and then subtracted from 1 to give the tolerance.

The tolerance indicates the proportion of variance in the dependent OMI variable (Y) that cannot be explained by the remaining three independent OMI variables (X_1, X_2 and X_3). The computational process involves the use of the regression models, detailed in Table S5. The results of the multicollinearity test are tabulated in Table S6.

For example, the tolerance value for deprivation (Table S6) indicates that about 50% of the variance in deprivation cannot be explained by the remaining three variables (instability, dependency and ethnic concentration). Similarly, the deprivation, dependency and ethnic concentration variables cannot explain 70% of the variance in the instability dimension of OMI.

The high tolerance values indicate that the four OMI variables are relatively unique and cannot be linearly predicted from one another with sufficient details.

b) Variance Inflation Factors (VIF):

The VIF equals to $\frac{1}{\text{Tolerance}}$ and evaluates the inflation in the variances of the parameter estimates due to the collinearities amongst the predictor or independent variables.

In general, variables with VIF values greater than 4 require further investigation to understand their relative contributions in the model and VIF values greater than 10 must have to be corrected using statistical techniques such as the Principal Component Analysis [6].

The VIF values for each of the tested variables are well below 4, indicating that there are insufficient collinearities amongst the remaining three predictor variables to inflate the parameter estimates. This confirms the conclusion from the evaluation of tolerance values that the variables are relatively unique to each other and, when added together in a regression model, should not demonstrate sufficient multicollinearity to bias the regression results.

As the results of the Pearson correlation coefficient and multicollinearity tests did not yield signs of notable correlation and multicollinearity amongst the variables, more sophisticated tests of multicollinearity, such as the evaluation of the eigenvalues to assess the relative contributions of the OMI variables in a regression model, were not necessary.

Table S5. The models used to generate the multicollinearity test statistics.

	Y	X_1	X_2	X_3
**Model 1:**	Instability	Deprivation	Dependency	Ethnic concentration
**Model 2:**	Deprivation	Instability	Dependency	Ethnic concentration
**Model 3:**	Dependency	Instability	Deprivation	Ethnic concentration
**Model 4:**	Ethnic concentration	Instability	Deprivation	Dependency

Table S6. Results of the multicollinearity test.

Model	Variable tested	R^2	Tolerance	VIF
1	Instability	0.260	0.740	1.352
2	Deprivation	0.458	0.542	1.844
3	Dependency	0.246	0.754	1.326
4	Ethnic concentration	0.453	0.547	1.827

References

1. Glazier RH, Gozdyra P, Kim M, Bai L, Kopp A, Schultz SE, Tynan AM. Geographic Variation in Primary Care Need, Service Use and Providers in Ontario, 2015/16. Toronto, ON: Institute for Clinical Evaluative Sciences; 2018.
2. USGS. Landsat Surface Reflectance-Derived Spectral Indices - Landsat Enhanced Vegetation Index. Availabe online: https://www.usgs.gov/land-resources/nli/landsat/landsat-enhanced-vegetation-index?qt-science_support_page_related_con=0#qt-science_support_page_related_con (accessed on December 20, 2019).
3. USGS. Landsat Surface Reflectance-Derived Spectral Indices - Landsat Normalized Difference Vegetation Index. Availabe online: https://www.usgs.gov/land-resources/nli/landsat/landsat-normalized-difference-vegetation-index?qt-science_support_page_related_con=0#qt-science_support_page_related_con (accessed on December 20, 2019).
4. USGS. Landsat Surface Reflectance-Derived Spectral Indices - Landsat Soil Adjusted Vegetation Index. Availabe online: https://www.usgs.gov/land-resources/nli/landsat/landsat-soil-adjusted-vegetation-index (accessed on December 20, 2019).
5. Mansfield, E.R.; Helms, B.P. Detecting multicollinearity. *The American Statistician* **1982**, *36*, 158-160.
6. Collinearity Diagnostics, Model Fit & Variable Contribution. Availabe online: https://cran.r-project.org/web/packages/olsrr/vignettes/regression_diagnostics.html (accessed on 15 Sept 2019).
